# Acute electroencephalography responses during incremental exercise in those with mental illness

**DOI:** 10.3389/fpsyt.2022.1049700

**Published:** 2023-01-12

**Authors:** C. V. Robertson, M. Skein, G. Wingfield, J. R. Hunter, T. D. Miller, T. E. Hartmann

**Affiliations:** ^1^School of Exercise Science, Sport and Health, Charles Sturt University, Bathurst, NSW, Australia; ^2^Western NSW Local Health District, Dubbo, NSW, Australia; ^3^Holsworth Research Initiative, La Trobe University, Bendigo, VIC, Australia

**Keywords:** mental illness, EEG, exercise, gamma, frontal alpha asymmetry

## Abstract

**Introduction:**

Depression is a mental illness (MI) characterized by a process of behavioral withdrawal whereby people experience symptoms including sadness, anhedonia, demotivation, sleep and appetite change, and cognitive disturbances. Frontal alpha asymmetry (FAA) differs in depressive populations and may signify affective responses, with left FAA corresponding to such aversive or withdrawal type behavior. On an acute basis, exercise is known to positively alter affect and improve depressive symptoms and this has been measured in conjunction with left FAA as a post-exercise measure. It is not yet known if these affective electroencephalography (EEG) responses to exercise occur during exercise or only after completion of an exercise bout. This study therefore aimed to measure EEG responses during exercise in those with MI.

**Materials and methods:**

Thirty one participants were allocated into one of two groups; those undergoing management of a mental health disorder (MI; *N* = 19); or reporting as apparently healthy (AH; *N* = 12). EEG responses at rest and during incremental exercise were measured at the prefrontal cortex (PFC) and the motor cortex (MC). EEG data at PFC left side (F3, F7, FP1), PFC right side (F4, F8, FP2), and MC (C3, Cz, and C4) were analyzed in line with oxygen uptake at rest, 50% of ventilatory threshold (VT) (50% VT) and at VT.

**Results:**

EEG responses increased with exercise across intensity from rest to 50% VT and to VT in all bandwidths (*P* < 0.05) for both groups. There were no significant differences in alpha activity responses between groups. Gamma responses in the PFC were significantly higher in MI on the left side compared to AH (*P* < 0.05).

**Conclusion:**

Alpha activity responses were no different between groups at rest or any exercise intensity. Therefore the alpha activity response previously shown post-exercise was not found during exercise. However, increased PFC gamma activity in the MI group adds to the body of evidence showing increased gamma can differentiate between those with and without MI.

## Introduction

Depression affects 300 million people worldwide and is the leading cause of disability in populations aged between 15 and 44 years (1). Depression is a mental illness (MI) characterized by a process of behavioral withdrawal whereby people experience symptoms which include sadness, anhedonia, demotivation, sleep and appetite change as well as cognitive disturbances and suicidality in extreme cases (2, 3). The diagnosis of depression can be problematic with the use of subjective questionnaires and symptomology that overlaps with other psychiatric diagnoses, making finding a biomarker for depression an important pursuit. The availability of an objective diagnostic tool could aid in providing more reliable distinction between depression and other mental disorders with overlapping symptoms, ultimately providing better prognosis with earlier treatment.

As a low cost, easy to administer and non-invasive technique, scalp electroencephalography (EEG) has been investigated as a diagnostic tool in depression (4–6). Patients with depression exhibit dysregulated, elevated oscillatory activity, specifically in the alpha frequency band (8–12 Hz) in the prefrontal cortex (PFC) (7, 8). There is currently no clear consensus regarding the functional meaning of alpha wave activity (9), having been shown to be present in cognitive (10), sensorimotor (11), emotional (12), and physiological aspects (13), although historically being an inverse marker of cortical activation (14). These alpha oscillations are also detected by fMRI and have been shown to be positively correlated in a cingulo-insular-thalamic network (15) suggesting alpha synchronization plays a key global role in top down network control (16) and that it is a marker of underlying neural processes (12). Dysfunctional neural processes occurring with depression include the disruption of the serotonin system and increases in alpha activity may reflect low arousal associated with low serotonergic activity (17) which may be related to mitochondrial dysfunction (18). In addition to their potential diagnostic utility, EEG-based biomarkers also have predictive value for patient responses to a variety of depression treatment options (19, 20). This provides scope for the development of personalized medicine in the form of individually tailored treatment options for patients with depression (21). Specifically, alpha power has been shown to reduce following transcranial alternating current stimulation as a therapeutic intervention for treatment of major depressive disorder (22, 23) and to be a predictor of responders to anti-depressant medications (17).

Frontal alpha asymmetry (FAA) is a commonly studied depression biomarker which measures the relative alpha band activity between the brain hemispheres at the frontal electrodes. According to the approach-withdrawal hypothesis, different affective responses are reflected by activity in different sides of the brain. Left sided frontal brain activity is related to approach type behaviors, whereas right sided frontal brain activity (higher alpha activity on the left-hand side) is related to withdrawal behaviors such as those experienced in depression (24, 25). It is the balance in the activation of these systems which is assumed to be reflected in FAA EEG activity. FAA has indeed been reported to be different in those with depression, with depressed patients having higher alpha power in the left hemisphere indicative of withdrawal behaviors (4, 6, 8, 25–27). The presence of abnormal frontal and alpha asymmetries in depressed patients supports the view that they represent state-independent markers of vulnerability to negative affect and depressive disorders (28). Depression symptoms such as anhedonia have also been shown to account for a significant portion of the relationship between FAA and lifetime major depressive disorder (25). Additionally, a decrease in right sided frontal activity has been shown to relate to a decrease in negative affect (29), while an increase in left PFC activity (i.e., less alpha activity) has been associated with positive affect (30).

A positive affective state and improvement in depressive symptoms are well documented following exercise in healthy individuals (31–33) and clinical populations with depression (34, 35). Exercise has been shown to improve symptoms of depression as measured by several validated psychological screening tools (36, 37). This change in affect has been found following a range of exercise intensities which includes low intensity [15–39% oxygen uptake reserve (%*VO_2_*R)], and short durations, from 7 to 35 min. (33). Research examining the change in EEG response to exercise in relation to mood has reported a change in affect post-exercise in conjunction with left FAA or changes in alpha power spectral density (38–40). These EEG responses suggest a link between EEG asymmetry and affective responses to exercise and may explain the mechanism for the positive affect associated with improved mood after an exercise bout.

While there is evidence examining the EEG response following an exercise bout, the EEG responses during exercise in those with MI has not yet been elucidated. There are few studies examining EEG responses during exercise and none yet examining responses in those with MI. Furthermore, it is not yet known if these EEG responses to exercise occur during exercise or only after completion of the exercise bout. One proposed theory, the Transient Hypofrontality theory suggests that changes in brain activity responses during exercise are representative of changes in the PFC which promote the anxiolytic effects of exercise (41). Further work has also shown changes in brain responses to incremental exercise that align with changes in affective responses (42), with higher intensities yielding lower affective responses. This study therefore aimed to measure EEG responses in both the PFC and motor cortex (MC) during exercise in those with MI and healthy controls, utilizing an incremental test to exhaustion which encompasses all exercise intensities. Our hypotheses were that the EEG response at rest would be different between groups with those with MI showing FAA with higher alpha activity in the left PFC. EEG changes during exercise were hypothesized to be similar between groups due to the affective responses previously attributed to exercise, with an increased left sided activity (less alpha activity) indicative of positive affective responses. This study was written in line with the STROBE guidelines for case-control studies (43).

## Materials and methods

### Participants

For this case-control experiment, the study cohort included 31 participants allocated into one of two groups; those undergoing management of a medically diagnosed mental health disorder (MI; *N* = 19); or reporting as apparently healthy (AH; *N* = 12). Participants were male and female, between 18 and 62 years of age, non-smokers, free from thyroid disease, stroke, head trauma, epilepsy, multiple sclerosis, Alzheimer’s disease, Parkinson’s disease, Huntington’s disease and other neuromotor disorders or psychotic symptoms and performing <150 min per week of moderate intensity exercise or equivalent. For participants in the MI group who were receiving medication as treatment, a stable dose for 6 months was required with no comorbidities associated with psychiatric illness. Prior to the commencement of the study, all participants were required to provide written and verbal consent following an outline of all procedures and measures. This study conformed to the Declaration of Helsinki and was approved by the Research in Human Ethics Committee at Charles Sturt University.

Prior to commencing exercise, participants were required to undergo a medical screening by their general practitioner (GP) and be deemed eligible for safe participation in the exercise study. Participants completed an adult pre-exercise screening system (APSS, Exercise and Sports Science Australia) and a broader health history questionnaire before commencing testing. Participants also completed the Kessler 10 Psychological Distress Scale (K-10) (44) and the Depression Anxiety and Stress Scale (DASS-21) (45) to ascertain severity of current symptoms. The K-10 provides a measure of psychological distress over the preceding 4 weeks with higher scores indicating greater distress. (46). The DASS-21 measures and discriminates between depression, anxiety and stress with a set of three self-report scales, the scores for each domain calculated by summing the relevant items (47).

### Procedures

#### Resting

Participants reported to the laboratory in allotted time slots across successive mornings in a fasted state. This study was part of a larger study examining inflammatory responses which required fasting blood measures (36). The testing session took approximately 1 h for each participant to complete. Height was measured using a wall-mounted stadiometer (Custom CSU, Bathurst, NSW, Australia) and weight was measured using digital scales (HW 150 K, A & D, Bradford, MA, USA). On arrival participants were instrumented with a 20 channel, 256 Hz, wireless EEG headset (B-Alert, ABM, CA, USA) described subsequently. Following this, participants sat still looking at a blank wall with their eyes open while 2 min of resting data were collected. Following resting measurements, participants were moved to the cycle ergometer for the incremental exercise test.

#### Incremental exercise

Participants were set up on an electronically braked cycle ergometer (LODE Excalibur sport, LODE BV, Groningen, Netherlands) to ensure they were comfortable and were in an appropriate cycling position. Participants were then fitted with a facemask (Hans Rudolph, Kansas City, MO, USA) connected to a rapid response gas analyzer (AEI Technologies, Pittsburgh, PA, USA) for the measurement of pulmonary gas exchange. The hairnet for the facemask was utilized to prevent the EEG strip from moving thereby reducing artifact. An incremental test to exhaustion was then performed commencing at 25 watts (W) and increasing by 25 W every minute until volitional exhaustion. Participants were asked to keep their cadence as consistent as possible.

### Measurements

#### Electroencephalography

Measurements for EEG strap size were taken; sagittal, coronal plane and circumferential measures were taken for correct strap size and EEG placement using the 10–20 international system (48). Alignment of the EEG strip was ensured by placing the strip at 10% of the coronal measure from the nasion to the inion for placement of FP1 and FP2. The scalp electrode impedance (kΩ) of all electrodes was maintained below 40 kΩ. Paired mastoid references were used and electrode and reference sites were cleaned and abraded prior to fitting. Data were sampled with a bandpass filter from 0.5 and 65 Hz (at 3 dB attenuation) obtained digitally with Sigma-Delta A/D converters as previously reported (49). Data were acquired wirelessly across a R F link *via* an RS232 interface. While EEG signals were recorded at all 20 electrode sites, data produced at the following sites were used for analysis; FP1, FP2, F3, F4, F7, F8, C3, Cz, and C4.

### Data analysis

#### Ventilatory parameters

All gas exchange data were exported into an excel spreadsheet and time averaged over 15 s. Peak oxygen consumption ($\dot{V}\,O_{2}$ peak) was determined by the highest 15 s average. The ventilatory threshold (VT) was determined using previously defined methods utilizing increases in ventilatory equivalent for oxygen ($\dot{V}\,E/\dot{V}\,O_{2}$) (50). The point of 50% VT was computed using linear regression. The EEG data were then aligned to these timespoints by averaging the final 15 s prior to each timespoints: 50% VT and VT.

#### EEG analysis

The EEG data were processed and analyzed using B-Alert lab (ABM, CA, USA). Each 15 s sample was visually inspected for artifact (Polyman, version 1.153.1065) and eye blink and muscle artifact was removed by the B-Alert decontamination algorithms. Decontaminated data were then Fast Fourier Transformed into power spectra and power spectral densities (PSD) were calculated for the following frequency bands; Alpha Slow (αS) (8–10 Hz), Alpha Fast (αF) (10–13 Hz), Beta (β), and Gamma (γ) (30–40 Hz). Prior to statistical analysis EEG channels were divided into regions as follows: PFC left side; FP1, F7, and F3; PFC right side; FP2, F8, and F4; and MC (C3, Cz, and C4). EEG analysis was undertaken for the 2 min of resting data and the final 15 s of each exercise intensity timespoints.

#### Statistical analysis

A sample size of 16 was predicted to achieve statistical power based on a large effect size and a *P*-value set at 0.05 (G Power, Germany). Participants with excess artifact were removed from the data analysis leaving a total of 21 (AH; *n* = 9 and MI; *n* = 12). EEG data were analyzed using a mixed effects model (PFC side × intensity × group) with significance set at *P* < 0.05 once residuals were checked for normality [Jamovi, (51)]. EEG data were entered in absolute values rather than by calculating a laterality index. Results are presented as means (±) standard deviation (SD).

## Results

### Participant characteristics

Participant physical characteristics and incremental exercise test outcomes are provided in Table 1. Compared to AH participants, MI participants were on average significantly younger (*P* < 0.05), had a greater body weight (*P* < 0.05) and body mass index (*P* < 0.01), and had a lower $\dot{V}\,O_{2}$ peak (*P* < 0.05). There was no significant difference in VT (as a % of $\dot{V}\,O_{2}$ peak) and in test length between groups.

**TABLE 1:** Physical characteristics of participants and incremental exercise test outcomes.

	AH (*n* = 9)		MI (*n* = 12)	
	**Mean**	**SD**	**Mean**	**SD**
Age (years)	47.78	5.63	39.67*	9.7
Height (m)	1.71	0.1	1.69	0.12
Weight (kg)	74.47	13.5	100.45*	28.82
BMI (kg⋅m^–2^)	22.87	3.53	35.16**	10.03
$\dot{V}\,O_{2}$ peak (ml⋅kg⋅min^–1^)	27.59	7.93	20.28*	6.41
VT at % $\dot{V}\,O_{2}$_*peak*_	74.31	19.21	82.01	14.2
Test length (s)	406	192	457	168

AH, apparently healthy; MI, mental illness; n, number of subjects; VT, ventilatory threshold.

*P < 0.05; **P < 0.01.

Participant mental health characteristics are provided in Table 2. Compared to AH participants, MI participants had significantly higher K-10 psychological distress scores (*P* < 0.05), DASS-21 depression (*P* < 0.05), anxiety (*P* < 0.01), and stress (*P* < 0.05) scores.

**TABLE 2:** Mental health characteristics of participants.

	AH (*n* = 9)		MI (*n* = 12)	
	**Mean**	**SD**	**Mean**	**SD**
**K-10**	13.9	4.02	18.9*	7.30
**DASS-21 scores**				
Depression	4	5.5	14.4*	15.5
Anxiety	1.67	2.55	8.00**	4.00
Stress	5.83	6.61	15.2*	11.4

AH, apparently healthy; MI, mental illness; n, number of subjects; K-10, Kessler 10 psychological distress scale; DASS-21, depression anxiety and stress scale. *P < 0.05; **P < 0.01.

### Prefrontal cortex EEG responses

#### Alpha slow

**FIGURE 1:**
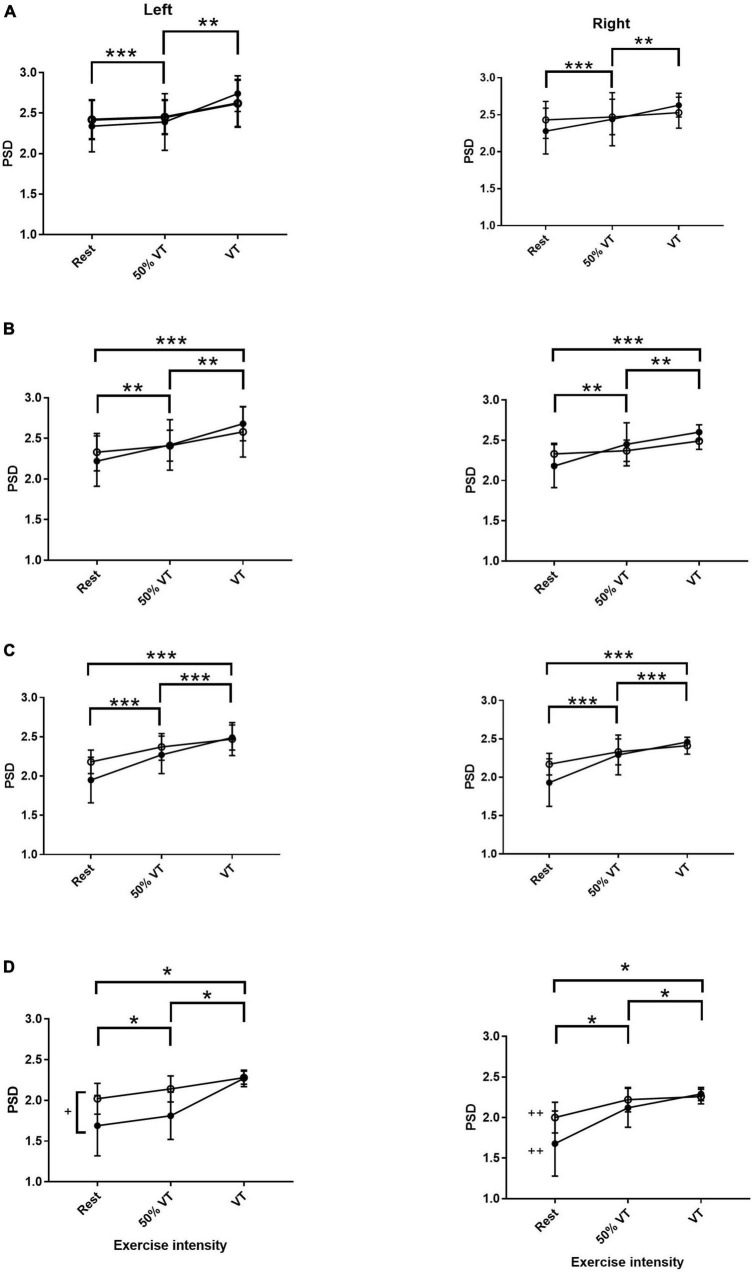
Power spectral density (PSD) responses in alpha slow **(A)**, alpha fast **(B)**, beta **(C)**, and gamma **(D)** within the prefrontal cortex in AH (●) and MI (▲) in the left and right hand sides. Significant differences across intensities; *P < 0.05, **P < 0.01, ***P < 0.001. + significant differences between groups (*P* < 0.05) and ++ significant differences between gamma left AH and MI (*P* < 0.05).

There was no effect of group or side in αS, however, there was an effect of intensity. Power spectral density in αS increased from rest to VT (*P* < 0.001) and from 50% VT to VT (*P* < 0.01) (see Figure 1 and Table 3). Activity levels at rest were higher in MI compared to AH on both left and right sides (2.43 ± 0.25 vs. 2.34 ± 0.32; 2.47 ± 0.24 vs. 2.29 ± 0.32) respectfully, however, this was not significant (*P* > 0.05).

**TABLE 3:** Electroencephalography (EEG) responses (mean ± SD) in AH and MI in the prefrontal cortex on the left and right hand sides at rest, 50% VT and VT.

		AH (*n* = 9)				MI (*n* = 12)			
		**Rest**	**50% VT**	**VT**		**Rest**	**50% VT**	**VT**	
Alpha slow	Left	2.34 (0.32)	2.39 (0.35)	2.74 (0.22)	### &&	2.42 (0.24)	2.45 (0.21)	2.62 (0.29)	### &&
	Right	2.28 (0.24)	2.44 (0.36)	2.63 (0.16)		2.43 (0.25)	2.47 (0.24)	2.53 (0.21)	
Alpha fast	Left	2.22 (0.31)	2.42 (0.31)	2.68 (0.21)	## $$ &&&	2.33 (0.23)	2.41 (0.19)	2.58 (0.31)	## $$ &&&
	Right	2.18 (0.32)	2.45 (0.32)	2.60 (0.11)		2.33 (0.22)	2.41 (0.19)	2.58 (0.31)	
Beta	Left	1.95 (0.29)	2.27 (0.24)	2.49 (0.16)	*** &&& ###	2.18 (0.15)	2.33 (0.17)	2.47 (0.21)	*** &&& ###
	Right	1.93 (0.31)	2.29 (0.26)	2.46 (0.02)		2.17 (0.14)	2.33 (0.17)	2.41 (0.11)	
Gamma	Left	1.69 (0.37)	1.81 (0.29)	2.27 (0.10)^∞^ ^α^ ^α^ ^α^	## &&	2.02 (0.19)	2.14 (0.16)	2.28 (0.08)	* ###
	Right	1.68 (0.40)	2.12 (0.24)	2.29 (0.08)		2.00 (0.19)	2.22 (0.15)	2.26 (0.09)	

Symbols denote difference from rest to 50% VT (*), rest to VT (^#^) and from 50% VT to VT (^&^) with level of significance set as number of symbols; one, (*P* < 0.05), two (*P* < 0.01), three (*P* < 0.001). Differences between sides denoted as (●) higher than left side, (∞) as lower than AH right side and (α) as lower than MI right side, with number of symbols denoting significance, as above.

#### Alpha fast

There was no effect of group or side in αF, however, PSD in αF showed a significant effect of intensity. There was a significant increase in AF from rest to 50% VT (*P* < 0.01) from 50% VT to VT (*P* < 0.01) and from rest to VT (*P* < 0.001) (see Figure 1 and Table 3).

#### Beta

There was only a significant effect of intensity in the β EEG response (*P* < 0.001) with β increasing significantly from rest to 50% VT (*P* < 0.001), from 50% VT to VT (*P* < 0.001) and from rest to VT (*P* < 0.001) (see Figure 1 and Table 3).

#### Gamma

There was a significant effect of group (*P* < 0.05), side (*P* < 0.02), and intensity (*P* < 0.001) in the γ response with both group x intensity and intensity x side interactions (*P* < 0.05). The γ response increased in the MI group from rest to 50% VT (*P* < 0.05) and from rest to VT (*P* < 0.001) and in the AH group from rest to VT (*P* = 0.01), from rest to 50% VT (*P* < 0.01) and from 50% VT to VT (< 0.01) (see Figure 1 and Table 3).

The left side γ activity in the MI group was significantly higher than in the left side AH group (*P* < 0.05). The AH response on the left side was significantly lower than both the AH right side (*P* < 0.05) and the MI right side (*P* = 0.001) (see Figure 1 and Table 3).

#### Motor cortex EEG responses

**FIGURE 2:**
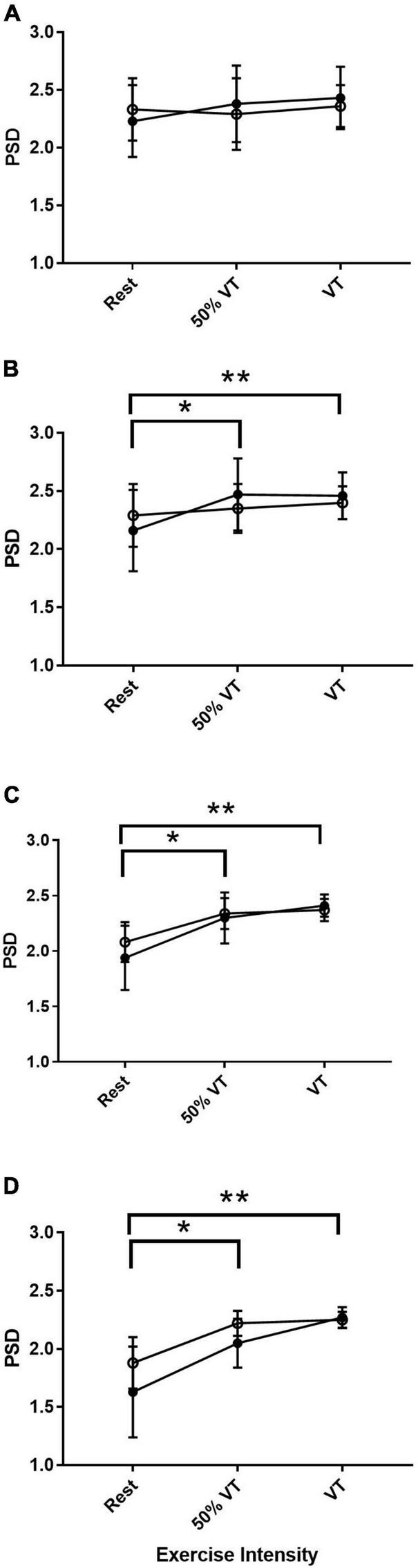
Power spectral density (PSD) responses in alpha slow **(A)**, alpha fast **(B)**, beta **(C)**, and gamma **(D)** within the motor cortex in AH (●) and MI (°). Significant differences between rest and intensities; *P < 0.05, **P < 0.01.

Across all bandwidths there was no significant effect of group in the EEG response in the MC. In the bandwidth αS, there were no significant differences across time points from rest, 50% VT and at VT (*P* > 0.05) (see Figure 2). In αF, β, and γ bandwidths, there was a significant effect of time (*P* < 0.05) with power spectral density increasing from rest to 50% VT (*P* < 0.05) and from rest to VT (*P* < 0.01) (see Figure 2 and Table 4).

**TABLE 4:** Electroencephalography (EEG) responses (mean ± SD) in AH and MI in the motor cortex at rest, 50% VT and VT.

	AH (*n* = 9)					MI (*n* = 12)		
	**Rest**	**50% VT**	**VT**		**Rest**	**50% VT**	**VT**	
Alpha slow	2.23 (0.31)	2.38 (0.33)	2.43 (0.27)		2.33 (0.24)	2.29 (0.31)	2.36 (0.18)	
Alpha fast	2.16 (0.35)	2.47 (0.31)	2.46 (0.20)	* ^##^	2.29 (0.27)	2.35 (0.21)	2.40 (0.14)	* ^##^
Beta	1.94 (0.29)	2.30 (0.23)	2.41 (0.10)	* ^##^	2.08 (0.18)	2.34 (0.14)	2.37 (0.10)	* ^##^
Gamma	1.63 (0.39)	2.05 (0.21)	2.27 (0.09)	* ^##^	1.88 (0.22)	2.22 (0.11)	2.25 (0.07)	* ^##^

Symbols denote difference from rest to 50% VT (*) and rest to VT (^#^) with level of significance set as number of symbols; one, (*P* < 0.05), two (*P* < 0.01), three (*P* < 0.001).

## Discussion

This study investigated the EEG responses during rest and incremental exercise to exhaustion in both those who were AH and those experiencing a MI. To our knowledge this is the first study to measure EEG responses during exercise in those with MI. Our main findings were that while resting αS and αF were both higher in those with MI compared to AH, this was not significantly so, and there was no difference between the left and right PFC suggesting that no FAA was present. From rest to exercise, each bandwidth increased significantly in both AH and MI (see Figures 1, 2) between at least two of the three intensities. There was a significant difference in the gamma response between groups with gamma being significantly higher on the left PFC in the MI group. The gamma response in the AH left PFC was significantly lower than both the AH right PFC and the MI right PFC. These gamma responses add to the evidence that gamma may be an indicator of differences between AH and those with MI.

### Frontal alpha asymmetry

While there is considerable evidence in favor of the alpha asymmetry hypothesis to identify depression (4, 6, 8, 24, 27), there is also building evidence disputing the effectiveness of alpha asymmetry in diagnosis of depression (52). In their meta-analysis the authors highlight some concerns with the FAA literature which may impact interpretations of the findings. They suggest that the conflicting findings across the FAA literature are due to the considerable heterogeneity across study samples (age, gender, symptom severity) impacting the FAA response. This lack of FAA is in line with our results and we note that our MI population were of mixed ages and genders, potential confounding factors. Other potential reasons for the lack of FAA may be due to the combined analysis of channels on the left and right side of the PFC, rather than at individual channel sites. Some previous research has only analyzed one electrode site rather than multiple across one region (4, 6). Although there are others who have combined electrode sites (8) as with our data. There are additional discrepancies depending on the use of eyes closed vs. eyes open measures and if participants are currently utilizing medication (27, 53).

There may, however, be alternating explanations. FAA has been shown to be a predictor of patient responses to the anti-depressant Fluoxetine (Prozac), with non-responders having overall greater resting activation of the right hemisphere asymmetry in the eyes open condition, with this difference being predominantly in the fast alpha range (53). The authors propose that the non-responders resemble patients who have depression with comorbid anxiety disorder, i.e., experiencing different forms of depression. Frontal asymmetry and the degree of activation on each side may therefore be linked to forms of depression, comorbid conditions, or represent a degree of treatment resistance to certain medications. This highlights the importance of EEG asymmetry as a potential marker for clinicians to identify which anti-depressants to use. Further to this, other mechanisms proposed include the relationship between EEG alpha power and brain-derived neurotrophic factor (BDNF) Met/Met polymorphism (54), an indicator of depression severity. A reduced secretion of BDNF may negatively affect functional connectivity in neuronal systems that generate alpha oscillations (54).

### Gamma response and depression

The use of gamma oscillations as a biomarker for major depression is an emerging topic (55), with considerable literature examining topics such as gamma responses in treatment resistant depression with ketamine (56), responses to selective attention tasks (57) and as a biomarker for major depressive disorder recurrence (58). Gamma activity is found in a multitude of high level cognitive tasks, such as sensorimotor integration (59), short term or working memory (60) and language tasks (61), however, there is an argument that there is a more elementary explanation for its presence (62). Merker and colleagues (62) propose that Gamma activity instead holds an infrastructural support role whereby it supports neural activity rather than being involved in each cognitive function. As part of their proposal they outline that gamma activity may be contributed to by cortical inhibitory interneurons interacting among themselves to aid in providing inhibitory activity. This activity is necessary for keeping single units from saturating, acting almost like an emergency brake for run-away excitation (62). What role this may play in MI is not yet known.

Changes in gamma responses to depression have been shown to occur in the hippocampus (63) and the anterior cingulate cortex (57) and to be suppressed in various brain sites by serotonin boosting anti-depressants in rats (64, 65). However, there is scarce data examining the PFC. In a comparison between healthy participants and those with major depression, those with major depression were shown to have higher gamma (30–40 Hz) responses in frontal and temporal regions (66) on both sides of the PFC. Further, major depression patients with implanted deep brain simulation showed a significant decrease of frontal gamma, with decreasing depressive symptoms also associated with a decrease in right frontal gamma (67). These data suggest that a depressive state is accompanied by a higher PFC gamma response, similar to our findings. Additionally, work examining non-linear EEG responses to depression have shown an increase in fractal dimensions in the gamma bandwidth in the PFC indicating more complex electrophysiological behavior (68). The authors propose that this pattern of brain activity may represent cognitive dysfunction in depressed patients, a theory supported by the cognitive deficits found within depression (63) that may be modulated by neurotransmitter responses (63). However, as with FAA, findings across gamma activity in those with depression are inconsistent (55).

### EEG response and exercise

All bandwidths increased significantly across time with increasing exercise intensity from rest to the point of VT. This increase in EEG activity with incremental exercise has been shown previously in an AH population (49). This finding suggests that without the presence of alpha asymmetry during exercise, that changes in FAA measured previously following exercise (39, 69) do not occur during exercise. However, we found no FAA at rest and therefore this may have confounded a lack of findings in FAA during exercise. Alpha asymmetry and affective responses to exercise have been shown to be influenced by fitness levels (39) with resting frontal asymmetry predicting affect only in a high-fit group, suggesting in order to achieve affective responses that are positive a level of fitness is required. The differences in fitness between the AH and MI groups in our study may therefore be problematic, however, it has also been shown that affective responses are variable in relation to exercise intensity preference (38) thus, potential affective responses and EEG responses may be heterogenous both within and between groups during incremental exercise. Greater relative frontal activation at rest has also been shown to predict a positive affect post-exercise following exercise at 70% $\dot{V}\,O_{2}$ max, suggesting that FAA pre-exercise is an important determinant of changes post-exercise in the EEG response (69). As we saw no FAA at rest, this may provide a reason why there were not EEG changes during exercise. Further to this, while there is considerable evidence looking at the affective responses to exercise in those with MI we do not know if individuals with MI have the same affective responses during exercise as AH populations or if they are the same magnitude.

The gamma response to exercise is not well characterized, with most studies exploring changes in alpha and beta bandwidths (70, 71). One study measuring EEG post-exercise found no changes in gamma activity post-exercise and no relation to affective response (38). While there were significant interactions in the gamma response at the group × side level in our data, these did not reach significance at the group × side × intensity level thus it is not possible to say there were differences in gamma responses across groups and sides at each timespoints, although gamma activity was higher in MI at rest and 50% VT. If gamma plays any role in affect is not yet known.

### Limitations

It is recognized that the measurement of EEG during exercise is problematic due to muscle artifact (72). Technology for the measurement of EEG has developed rapidly allowing for the use of wireless technologies, significantly reducing artifact. Steps were also taken to ensure that participants kept as still as possible during the trial and that while we measured oxygen uptake, this was done with a mask not a mouthpiece as is often used which can add to muscle artifact. The headset used for attaching the mask to the face of the participants also aided in keeping the EEG strap still. Due to levels of artifact, the EEG response beyond the VT was not reportable. Overall, this is a consideration for this study. However, it should be noted that many studies have been previously published successfully showing EEG responses to exercise (70, 71).

Some considerations for the lack of FAA are that the period of 2 min eyes open resting EEG is a relatively short time, which may explain the discrepancies in resting EEG between AH and MI, however, this time period has been used previously in EEG research (73). While we achieved statistical power as determined by G Power calculations, the presence of artifact meant missing data which reduced our statistical power and therefore may have impacted us finding alpha asymmetry. Other confounding variables consist of the handedness of individuals and the age. Both of these variables have been shown to impact EEG activity. There has previously been reported an association between right hemisphere activity and negative affect *via* an association between EEG activity and non-right handedness (74), as well as a rightward bias in FAA with age (75).

## Conclusion

The EEG response to exercise in those with MI and AH controls showed a similar response to what has been shown previously in both the PFC and MC. There were no differences in alpha responses between groups at rest or during exercise suggesting that no resting FAA existed between groups and that the changes in alpha activity, indicative of improved affect, previously found post-exercise (39, 69) do not occur during exercise. FAA has been found across MI populations with depression (4, 6, 24–26) however, there is also evidence disputing the effectiveness of alpha asymmetry in diagnosis of depression due to non-generalizable results (52). The mechanisms behind differences in FAA are not yet fully elucidated but may include age, gender and symptom severity (52) forms of depression (76) and medication responses (53) and these factors may explain our lack of FAA findings.

The gamma response in those with MI was significantly higher than in the AH group, suggesting the usefulness of gamma responses to act as a biomarker for MI. The differences between groups in gamma activity may be due to altered neural dysfunction in those with depression. Gamma has been shown to decrease following use of neurotransmitter medication, thus, this altered neural function may also be indicative of changes in neurotransmitter responses found in depression (63, 64) which contribute to the emotional and cognitive disturbances found in depression (77).

## Data availability statement

The raw data supporting the conclusions of this article will be made available by the authors, without undue reservation.

## Ethics statement

The studies involving human participants were reviewed and approved by the Charles Sturt University Human Research Ethics Committee. The patients/participants provided their written informed consent to participate in this study.

## Author contributions

CVR contributed to the study design, data collection, data analysis, and manuscript writing. MS, GW, JRH, and TDM contributed to the data collection and manuscript review. TEH contributed to the study design, data collection, and manuscript review. All authors contributed to the article and approved the submitted version.
